# Optimal duration of perioperative antibiotics in radical cystectomy and urinary diversion: a systematic review and meta-analysis

**DOI:** 10.1007/s00423-025-03943-x

**Published:** 2026-01-15

**Authors:** Tarek Mohamed, Baha’ Aldeen Bani Irshid, Hamza Elhashamy, Mohammad Ghassab Deameh, Ahmed Hassab El-Naby, Mohamed Ramez

**Affiliations:** 1 Urology Department, United Lincolnshire Hospitals NHS Trust, Lincoln, UK; 2 Princess Basma Teaching Hospital, Irbid, Jordan; 3 Faculty of Medicine, Assiut University, Assiut, Egypt; 4 Prince Hamza Hospital, Amman, Jordan; 5 Urology Department, Faculty of Medicine, Assiut University, Assiut, Egypt; 6 University of Texas MD Anderson Cancer Center, Houston, TX USA

**Keywords:** Perioperative antibiotics, Prophylaxis, Radical cystectomy, Urinary diversion

## Abstract

**Purpose:**

To evaluate the impact of extended versus nonextended perioperative antibiotic prophylaxis (PAP) on reducing postoperative complications and hospital stays in patients undergoing radical cystectomy and urinary diversion.

**Methods:**

We conducted this systematic review and meta-analysis in accordance with the PRISMA guidelines. A comprehensive literature search was conducted across PubMed, Scopus, Web of Science, and the Cochrane Library for studies comparing short-term (≤ 24 h) and extended (≥ 24 h) PAP in patients undergoing radical cystectomy and urinary diversion. The primary outcomes were surgical site infections (SSIs), urinary tract infections (UTIs), and length of hospital stay. The statistical analysis was performed via RevMan version 5.3. The results are presented as risk ratios (RRs) and mean differences (MDs). Results are presented as risk ratios (RRs) and mean differences (MDs). The quality of evidence was assessed using the GRADE methodology.

**Results:**

A total of 214 studies were screened. Four studies involving 680 patients were included. No significant differences were detected between short-term and extended PAP in terms of SSIs (RR = 0.71 [95% CI 0.43–1.17]; *P* = 0.18]), febrile UTIs (RR = 1.19 [95% CI 0.91–1.56]; *P* = 0.20]), or length of hospital stay (MD = 0.76 days [95% CI [-2.72, 4.25]; *P* = 0.67]).

**Conclusion:**

No significant difference was observed between 24-h and extended PAP for reducing postoperative complications after radical cystectomy and urinary diversion. Short-term PAP is a reliable and effective strategy and is recommended as the standard practice for reducing antimicrobial resistance and improving postoperative outcomes.

## Introduction

Radical cystectomy (RC) is the cornerstone for the management of muscle invasive bladder cancer. It is also crucial for the treatment of high-risk and treatment-refractory nonmuscle invasive bladder cancer [1]. Ileal conduit urinary diversion is still the gold standard because it is technically simple to perform and is frequently chosen for older or comorbid patients because of its shorter recovery period, minimal bowel requirements for reconstruction, and decreased risk of complications [2]. Despite these benefits, early and late complications—such as ureteral obstruction, urinary tract infections, parastomal hernias, and stomal stenosis—remain prevalent, with average rates of 45% at 5 years [3].

Complications are common following RC since they involve simultaneous surgery of the urinary tract, digestive tract, and lymph nodes. The morbidity linked to RC is caused primarily by urinary tract infections (UTIs) and urosepsis, which occur at 30-day rates ranging from 28% to 60% [4]. Additionally, a five-year follow-up study of 66 patients who underwent RC and ileal conduit urinary diversion reported bacterial colonization and wound infection rates of 97% and 21.2%, respectively [5].

Perioperative antibiotic prophylaxis (PAP) is essential for reducing postoperative infections, such as surgical site infections (SSIs) and UTIs. The guidelines from the American Urological Association and the European Association of Urology recommend that the duration of PAP should not exceed 24 h postoperatively. AUA recommends that antibiotics be taken in a single dose of cefazolin. Alternative agents include single-dose clindamycin combined with an aminoglycoside, a second-generation cephalosporin, or an aminopenicillin with a β-lactamase inhibitor and metronidazole. The EAU recommended that the choice of antibiotic should be based on local bacterial resistance patterns and patient-specific factors.

A recent randomized clinical trial published in October 2024 compared 24-hour PAP to extended PAP in patients who underwent RC with urinary diversion. The trial revealed that 24-hour PAP was not inferior to extended PAP in preventing SSIs within 90 days, supporting the above guidelines to [6] prevent the development of antimicrobial resistance. However, there is a lack of adherence to guidelines and a high degree of heterogeneity in the antibiotic prophylaxis regime [7].

We are conducting the first meta-analysis to analyze the outcome of 24-hour PAP in reducing postoperative complications in radical cystectomy and urinary diversion, which can support more evidence and changes in daily practice.

## Methods

We conducted this systematic review and meta-analysis in accordance with the PRISMA (Preferred Reporting Items for Systematic Reviews and Meta-Analyses) guidelines [8]. The study protocol was registered on PROSPERO (CRD42024617744).

### Search strategy and inclusion criteria

A comprehensive search of PubMed, Scopus, Web of Science, and the Cochrane Library was conducted from inception to the present for studies published in English. We used Medical Subject Headings (MeSH) and keywords to identify randomized controlled trials (RCTs) and comparative observational studies. The search focused on adult patients (≥ 18 years) undergoing radical cystectomy with urinary diversion who received either short-term (≤ 24 h) or extended (> 24 h) PAP. Included studies had to report on at least one of the following outcomes: surgical site infections (SSIs), urinary tract infections (UTIs), or length of hospital stay. Two reviewers independently screened titles, abstracts, and full-text articles, with discrepancies resolved by a third reviewer.

#### Data extraction and quality assessment

Data extraction was conducted via a standardized form to ensure consistency and completeness. Two reviewers independently extracted data, including study characteristics (e.g., design, population size, and setting), participant demographics (e.g., age, sex, comorbidities & operative outcomes), details of the intervention (e.g., type, dose, and duration of antibiotics), and reported outcomes (e.g., SSI rates, UTIs, and length of hospital stay).

The risk of bias for the included studies was assessed separately on the basis of their design. For RCTs, the Cochrane risk of bias tool [9] was used, whereas the Newcastle‒Ottawa Scale [10] was used for non-RCTs. These assessments were independently conducted by two reviewers (MR, TM), with disagreements resolved through consensus or input from a third reviewer (HE).

#### GRADE evidence assessment

The overall quality of evidence for each primary outcome (SSIs, febrile UTIs, length of hospital stay) was assessed using the Grading of Recommendations, Assessment, Development, and Evaluations (GRADE) approach. The evidence was graded as high, moderate, low, or very low based on study design, risk of bias, inconsistency, indirectness, and imprecision.

#### Statistics

Data analysis was conducted using RevMan version 5.3. For dichotomous outcomes (SSIs, febrile UTIs), the Risk Ratio (RR) with a 95% confidence interval (CI) was calculated. For continuous outcomes (length of hospital stay), the Mean Difference (MD) with a 95% CI was calculated. A P-value of < 0.05 was considered statistically significant. Heterogeneity was assessed using the chi-square test and the I² statistic. A random-effects model was used for analysis if significant heterogeneity (I² >50% or *P* < 0.1 for chi-square test) was detected; otherwise, a fixed-effects model was applied. Subgroup analyses (e.g., plain vs. liposomal bupivacaine) were conducted where appropriate to explore potential sources of heterogeneity.

## Results

**Fig. 1:**
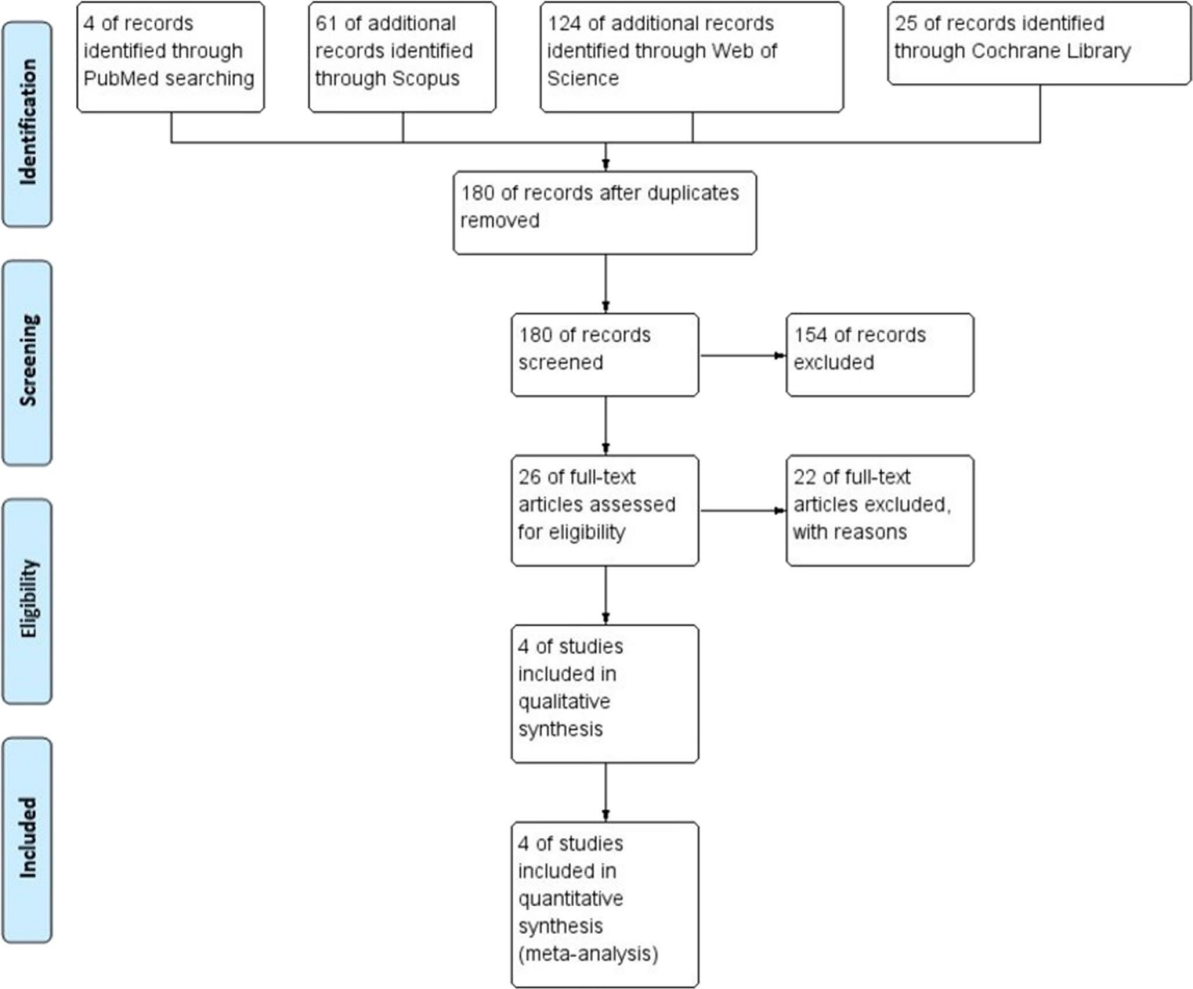
PRISMA flow chart of the meta-analysis

**Fig. 2:**
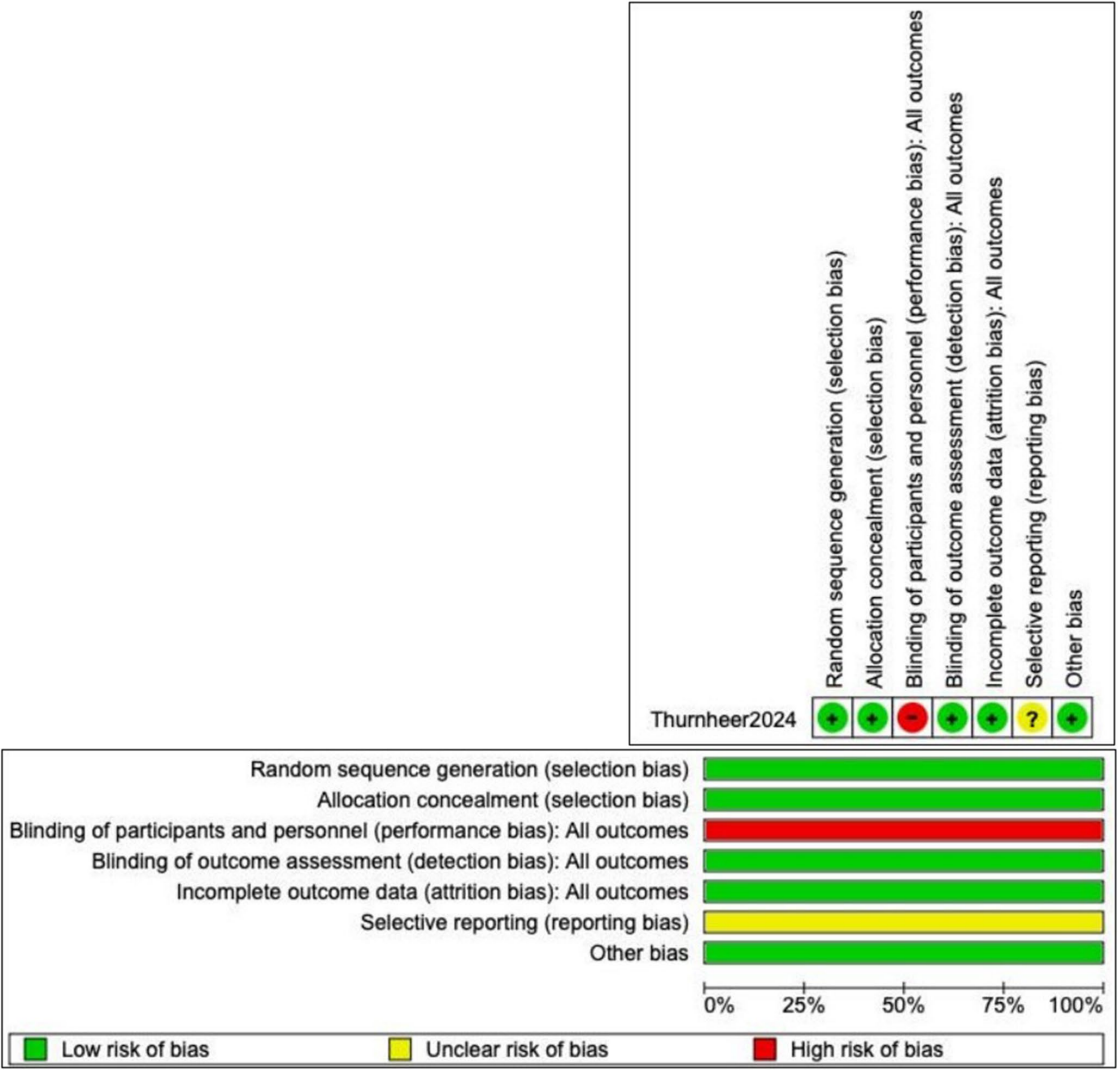
Risk of bias summary: review authors’ judgments about each risk of bias item for included randomized controlled trials

Our initial search yielded 214 studies, which were reviewed by two authors. Twenty-six full-text articles were assessed for eligibility, and of these, 4 studies [6, 11–13] with a total of 680 patients were included in the systematic review and meta-analysis. The PRISMA flow chart is presented in Fig. 1. Table 1 outlines the design and main results of the included studies, while Table 2 presents the baseline characteristics of the study populations. The results of the risk of bias assessment are shown in Fig. 2; Table 3. A summary of findings using the GRADE framework is presented in Table 4.

**Table 1:** Summary of included studies

ID	Study Design	Population	Intervention	Comparator	Outcomes	Key Findings	Type of Antibiotics Used
Hara 2008	Prospective Cohort	77 patients undergoing RC with ileal conduit for bladder cancer.	1-day AMP (piperacillin administered pre-, intra-, and postoperatively for 1 day).	3-day AMP (piperacillin for 3 days, same schedule).	SSIs, UTIs, pneumonia, ileus, and other complications within 30 days of surgery.	No significant difference in infection or complication rates between groups. One-day AMP was equally effective and reduced overall antibiotic exposure.	Piperacillin (2 g administered every 3 h intraoperatively, and every 12 h postoperatively for extended group).
Kim 2018	Retrospective Cohort	287 patients undergoing RC with orthotopic neobladder at a tertiary center in South Korea.	Short-term AMP (24-hour cefotetan, second-generation cephalosporin).	Long-term AMP (25 days: combination IV and oral antibiotics).	Febrile UTIs, bacteriuria, SSIs, resistant organisms, length of hospital stay, Clostridium difficile-associated diarrhea (CDAD).	Short-term AMP significantly reduced SSIs, and hospital stays, with no difference in febrile UTIs. Long-term AMP increased resistant infections.	Cefotetan (2nd-gen cephalosporin) for short-term. Long-term regimens: 3rd-gen cephalosporins, fluoroquinolones, trimethoprim-sulfamethoxazole, metronidazole.
Numao 2020	Retrospective Cohort	123 patients undergoing RC with ileal conduit or neobladder urinary diversion.	Intraoperative-only AMP (cefmetazole administered preoperatively and every 3 h intraoperatively).	Extended AMP (cefmetazole intraoperatively + cefotiam for up to 3 days post-op).	UTIs, SSIs, other infections, risk factors for infectious complications.	No significant difference in infection rates between groups. Shorter AMP minimized antibiotic exposure without increasing IC rates.	Cefmetazole (2nd-gen cephalosporin) intraoperatively; cefotiam (2nd-gen cephalosporin) for extended AMP. Levofloxacin (500 mg oral) during stent removal.
Thurnheer 2024	Randomized Clinical Trial	193 patients undergoing RC with urinary diversion (ileal conduit or neobladder).	24-hour AMP (antibiotics administered preoperatively, intraoperatively, and for 24 h post-op).	Extended AMP (antibiotics continued until catheter/stent removal; median 8 days).	SSIs, febrile UTIs, adverse events, and length of hospital stay.	24-hour AMP was noninferior to extended AMP for preventing SSIs, but extended AMP showed slightly lower febrile UTI rates (not significant). Shorter AMP reduced antibiotic exposure.	Third-generation cephalosporin (e.g., ceftriaxone) or cefuroxime, depending on local protocol.

**Table 2:** Baseline characteristics of included studies

ID	Sample Size		Age (years) Mean (SD)		Gender (Male) *n* (%)		BMI* (kg/m²) Mean (SD)		Diabetes *n* (%)		Hypertension *n* (%)		Blood Loss* (milliLiters) Mean (SD)		Operative Time* (minutes) Mean (SD)		Neoadjuvant Chemotherapy *n* (%)	
	Non-Extended	Extended	Non-Extended	Extended	Non-Extended	Extended	Non-Extended	Extended	Non-Extended	Extended	Non-Extended	Extended	Non-Extended	Extended	Non-Extended	Extended	Non-Extended	Extended
Hara 2008	33	44	66.9 (2.45)	64.8 (8.55)	23 (69.7%)	35 (79.5%)	20.9 (2.35)	21.7 (2.13)	5 (15.2%)	6 (13.6%)	9 (27.3%)	9 (20.5%)	NA	NA	NA	NA	4 (12.1%)	2 (4.5%)
Kim 2018	102	185	62.9 (10.5)	62.6 (10.4)	91 (89.2%)	159 (85.9%)	24.2 (3.3)	24.4 (2.9)	19 (18.7%)	32 (17.3%)	NA	NA	532.9 (300.7)	728.2 (445.8)	293.7 (47.2)	313.5 (57.2)	NA	NA
Numao 2020	58	65	65.33 (8.36)	67.67 (12.13)	42 (72.4%)	50 (76.9%)	23 (3.04)	22.33 (3.79)	4 (7%)	9 (14%)	NA	NA	913.33 (547.36)	1140 (743)	466.67 (98.83)	496.67 (136.47)	30 (52%)	20 (31%)
Thurnheer 2024	95	98	69.73 (10.31)	68.6 (11.06)	66 (69.5%)	68 (69.4%)	26.07 (4.14)	25.77 (4.51)	11 (11.6%)	9 (9.2%)	36 (37.9%)	35 (35.7%)	NA	NA	NA	NA	24 (26.4%)	28 (29.5%)

**Table 3:** Newcastle-Ottawa scale for risk of bias assessment of included studies

Study	Selection	Comparability	Outcome	Quality score
Hara 2008	★★★	★	★★★	7
Kim 2018	★★★	★★	★★★	8
Numao 2020	★★★	★★	★★★	8

Good quality: 3 or 4 stars (★) in selection domain AND 1 or 2 stars in comparability domain AND 2 or 3 stars in outcome domain; Fair quality: 2 stars in selection domain AND 1 or 2 stars in comparability domain AND 2 or 3 stars in outcome/exposure domain; Poor quality: 0 or 1 star in selection domain OR 0 stars in comparability domain OR 0 or 1 stars in outcome/exposure domain

**Table 4:** Summary of findings (GRADE)

Outcome	No. of Patients (Studies)	Risk of Bias	Inconsistency	Indirectness	Imprecision	Quality of Evidence	Anticipated absolute effects (95% CI) - Risk with Extended PAP vs. Short-term PAP
Surgical Site Infections	680 (4 studies)	Serious¹	Not serious	Not serious	Serious²	**Low**	**RR 0.71** (0.43 to 1.17). 3 fewer SSIs per 100 patients (from 6 fewer to 2 more).
Febrile UTIs	680 (4 studies)	Serious¹	Not serious	Not serious	Not serious	**Moderate**	**RR 1.19** (0.91 to 1.56). 2 more febrile UTIs per 100 patients (from 1 fewer to 6 more).
Length of Hospital Stay	680 (4 studies)	Serious¹	Very serious³	Not serious	Serious²	**Very Low**	**MD 0.76 days longer** (-2.72 shorter to 4.25 longer). Evidence is very uncertain.

CI: Confidence Interval; RR: Risk Ratio; MD: Mean Difference

¹ Downgraded due to risk of bias in non-randomized studies

² Downgraded for wide confidence intervals crossing the line of no effect

³ Downgraded for very high statistical heterogeneity (I²=97%)

### SSIs

**Fig. 3:**
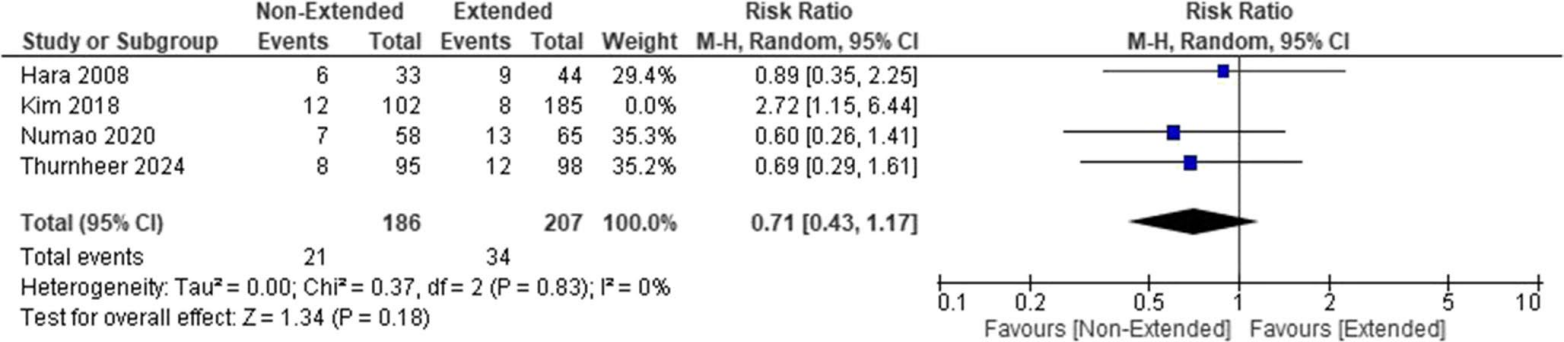
Forest plot showing SSIs

The analysis comparing short-term and extended PAPs revealed no significant differences in the risk of SSIs between the two approaches. The pooled risk ratio (RR) was 0.71 (95% CI [0.43–1.17]; *P* = 0.18). A sensitivity analysis was conducted by excluding Kim 2018 [12] to address heterogeneity, which did not change the significance of the results (Chi-square *P* = 0.83, I² = 0%). (Fig. 3)

### Febrile UTI

**Fig. 4:**
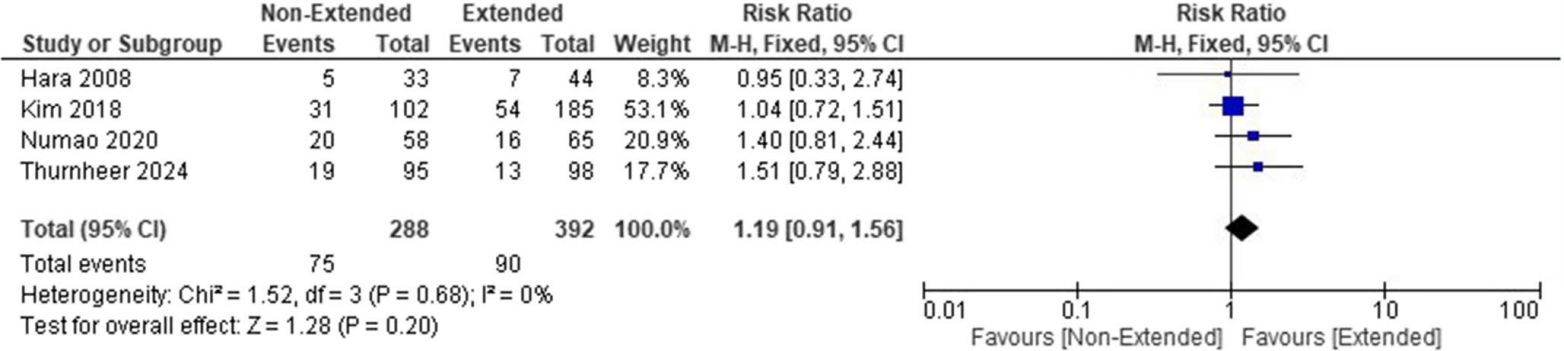
Forest plot showing Febrile UTIs

Evaluating febrile UTIs between short-term and extended PAPs revealed no statistically significant difference in risk between the two groups. The pooled risk ratio (RR) was 1.19 (95% CI [0.91–1.56]; *P* = 0.20). The studies were homogenous (Chi-square *P* = 0.68, I² = 0%). (Fig. 4)

### Hospital stay

**Fig. 5:**
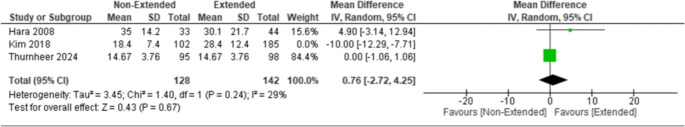
Forest plot showing Hospital stay without complications

**Fig. 6:**
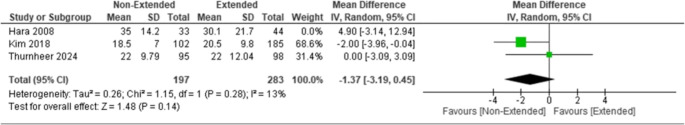
Forest plot showing Hospital stay with complications

Two analyses were conducted to evaluate the length of hospital stay. The first analysis, which excluded patients with postoperative complications (febrile UTIs or SSIs), found no significant difference between the short-term and extended PAP groups. The pooled mean difference (MD) was 0.76 days (95% CI [-2.72, 4.25]; *P* = 0.67), with low heterogeneity (I² = 29%) (Fig. 5). The second analysis, which included all patients, also showed no statistically significant difference. The pooled MD was − 1.37 days (95% CI [-3.19, 0.45]; *P* = 0.14), with low heterogeneity (I² = 13%) (Fig. 6). These findings indicate that the duration of antibiotic prophylaxis did not significantly impact the length of postoperative hospitalization.

## Discussion

Radical cystectomy with urinary diversion is the recognized standard treatment for patients with muscle-invasive bladder cancer [14]. Among the various forms of urinary diversion, the ileal conduit is the most frequently utilized because of its cost-effectiveness, clinical reliability, and favorable long-term outcomes [15]. However, complications such as urinary tract infections, sepsis, and surgical site infections are major contributors to morbidity during the inpatient recovery period and within one month following surgery [16].

The effect of prophylactic antibiotic duration prior to surgery on outcomes in radical cystectomy with urinary diversion remains inconclusive. To bridge this gap in the literature, we performed this meta-analysis to offer a more detailed evaluation of its potential impact.

Despite the importance of reducing postoperative infections, the establishment of an optimal antibiotic prophylaxis regimen for radical cystectomy remains elusive [17]. Several factors contribute to this lack of consensus, including the highly variable practices of antibiotic prophylaxis in RCs, inconsistent adherence to published guidelines, and the fact that most knowledge regarding antibiotic prophylaxis, particularly in cases involving ileal conduit urinary diversion, is derived from retrospective case series [13, 18].

This meta-analysis highlights critical findings regarding the role of perioperative antibiotic prophylaxis duration in patients undergoing radical cystectomy with urinary diversion. Our study evaluated the impact of prophylactic antibiotic duration on surgical site infections, febrile urinary tract infections and the duration of postoperative hospitalization following urinary diversion procedures. Contrary to the hypothesis that extended PAP could reduce postoperative complications, the analysis revealed no statistically significant differences between short-term and extended PAP in terms of the rates of SSIs, febrile UTIs, or the duration of postoperative hospitalization.

With increasing healthcare costs and adverse events, there is a growing call for the use of antibiotics when only medically necessary. It is also important to maintain the narrowest spectrum of activity and the shortest possible duration. Through a literature search, we could not find high-quality evidence to support the use of multiple doses of antibiotics. In addition, multiple studies have not shown any benefit of prolonged antibiotic use [19, 20]. Additionally, recent guidelines recommend that only a single dose of preoperative antibiotics be used without any exception for any type of surgical procedure [21].

Importantly, continuation of antibiotics beyond a single dose has also been associated with an approximately 4.5% risk of clostridial infection [22]. Additionally, prolonged antibiotic use has been significantly associated with antibiotic resistance without even decreasing the risk of SSI [23].

The results of this meta-analysis are consistent with findings from previous studies [11, 12], further supporting the conclusion that extending the duration of PAP does not provide substantial benefits in preventing infectious complications following RC with urinary diversion. These findings support the increasing consensus that short-term PAP is both safe and effective for patients undergoing RC, demonstrating comparable outcomes to extended PAP in preventing SSIs and febrile UTIs and influencing the postoperative length of stay. From a clinical perspective, reducing antibiotic exposure through short-term PAP aligns with global initiatives to mitigate antimicrobial resistance and minimize the risk of complications associated with prolonged antibiotic use.

An important clinical factor not specifically addressed in the included studies is the presence of ureteral stents, which are commonly used in urinary diversion and are known to be a nidus for infection. The management of PAP in stented patients, particularly around the time of stent removal, represents a gap in the literature. Future trials should aim to stratify patients based on stent usage or design protocols that specifically address stent-related infectious risks, as this may influence the optimal duration of antibiotic coverage.

This meta-analysis has limitations. The primary limitation is the heterogeneity across the included studies regarding the types of antibiotics used, the definitions of infectious complications, and the duration of “extended” prophylaxis, which ranged from 3 days to over 25 days. This clinical heterogeneity, combined with the statistical heterogeneity observed in the hospital stay outcome, suggests that the results should be interpreted with caution. Additionally, the inclusion of observational studies increases the risk of bias. Future research should focus on large, multicenter RCTs with standardized antibiotic protocols and outcome definitions to provide higher-quality evidence.

## Conclusions

This study demonstrated that, compared with short-term PAP, extending the duration of PAP does not significantly improve postoperative outcomes in patients undergoing radical cystectomy. Short-term PAP has proven to be a reliable and effective strategy, supporting its recommendation as the standard practice to reduce antibiotic exposure while maintaining optimal patient outcomes.

## Data Availability

No datasets were generated or analysed during the current study.
